# Supplementary material for: DNA Demethylation of Myogenic Genes May Contribute to Embryonic Leg Muscle Development Differences between Wuzong and Shitou Geese
Source: Int J Mol Sci. 2023 Apr 13;24(8):7188. doi: 10.3390/ijms24087188 (PMC10138404; doi:10.3390/ijms24087188)
Supplement: Supplementary file 1 [file ijms-24-07188-s001.zip › Supplementary Material.pdf]

Supplementary Material

DNA demethylation of myogenic genes may contribute to embryonic leg muscle development differences between Wuzong and Shitou geese

Xumeng Zhang[#,*], Yong Li[#], Chenyu Zhu, Fada Li, Zhiyuan Liu, Xiujin Li, Xu Shen, Zhongping Wu, Mengsi Fu, Danning Xu , Yunbo Tian and Yunmao Huang[*]

College of Animal Science & Technology, Zhongkai University of Agriculture and Engineering, Guangzhou 510225, China

# These authors contribute equally to the manuscript

[*] Correspondence:

Corresponding Author: Yunmao Huang, E-mail: huangyunmao@zhku.edu.cn;

Xumeng Zhang,E-mail: zhangxumeng@zhku.edu.cn.

1 Supplementary Data

Supplementary Data1. Detailed scripts of WGBS and RNA-seq analysis of this study have been provided in Supplementary Data.

2 Supplementary Figures and Tables

2.1 Supplementary Tables

Supporting information captions:

Table S1. Data filtering and aligning information of WGBS

Table S2. Distribution of CG, CHG and CHH methylation of the two goose breeds in gene, genome and transposable element regions.

Table S3. GO enrichment analysis information of differentially methylated genes (CG) between goose breeds at same stage.

Table S4. The methylation level of CG cites of *MyoD1* gene (upstream 2k, genebody and downstream 2k). The 13 CpGs analyzed by pyrosequencing are highlighted in yellow.

Table S5. The Pyrosequencing results of 13 methylation sites within the promoter region of *MyoD1*.

Table S6. The primers for RT-qPCR

Table S7. Design of pyrosequencing primers.

2.2 Supplementary Figures

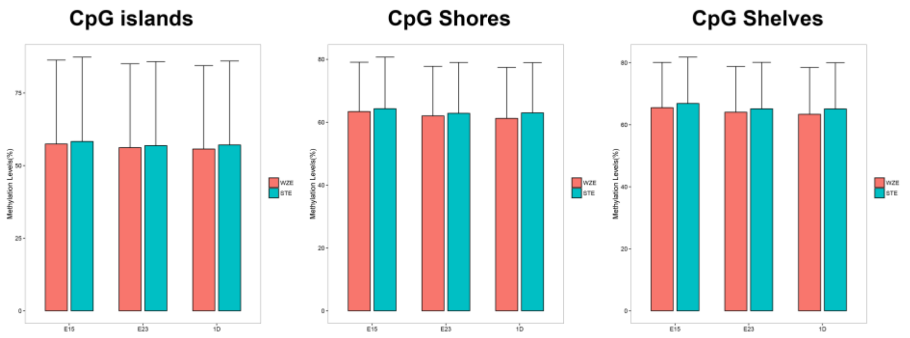

Figure S1. DNA methylation comparison of CpG islands, shelves and shores. The definition of CpG island is: CG content>50%, length>200 bp, observed CpG/expected

CpG>0.6. CpG shores refers to the area located within 2 kb upstream and downstream of CpG island; CpG shelves refers to the area located within 2 kb upstream and downstream of CpG shores (n=3).

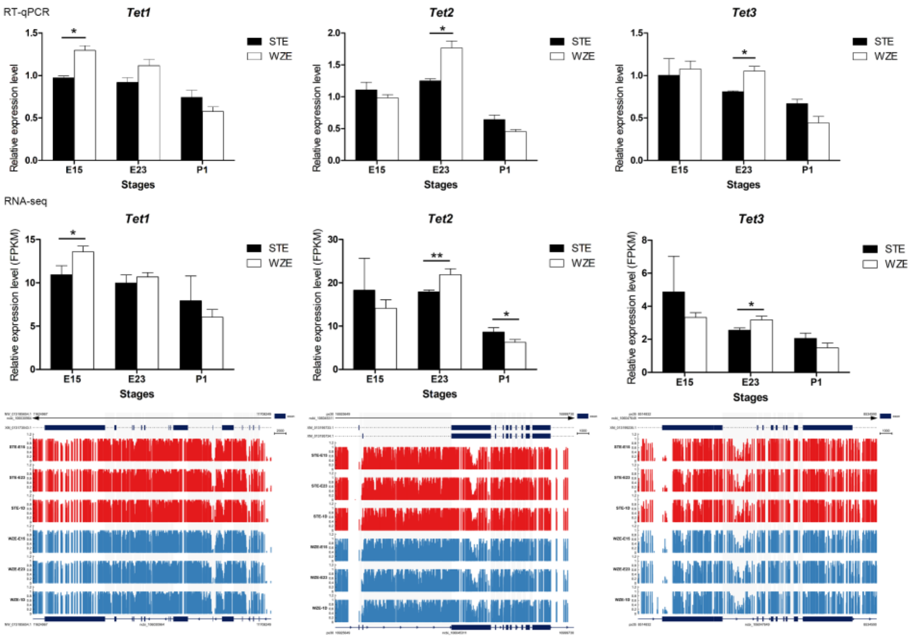

Figure S2. Gene expression patterns and DNA methylation level of *Tet1*, *Tet2* and *Tet3*. RT-qPCR and RNA-seq gene expression patterns and of *Tet1*, *Tet2* and *Tet3* in STE and WZE during E15–P1 were analyzed (n=3). DNA methylation level of *Tet1*, *Tet2* and *Tet3* in WGBS data was illustrated by IGV software in STE and WZE during E15–P1 (n=3). *$p < 0.05$; **$p < 0.01$.

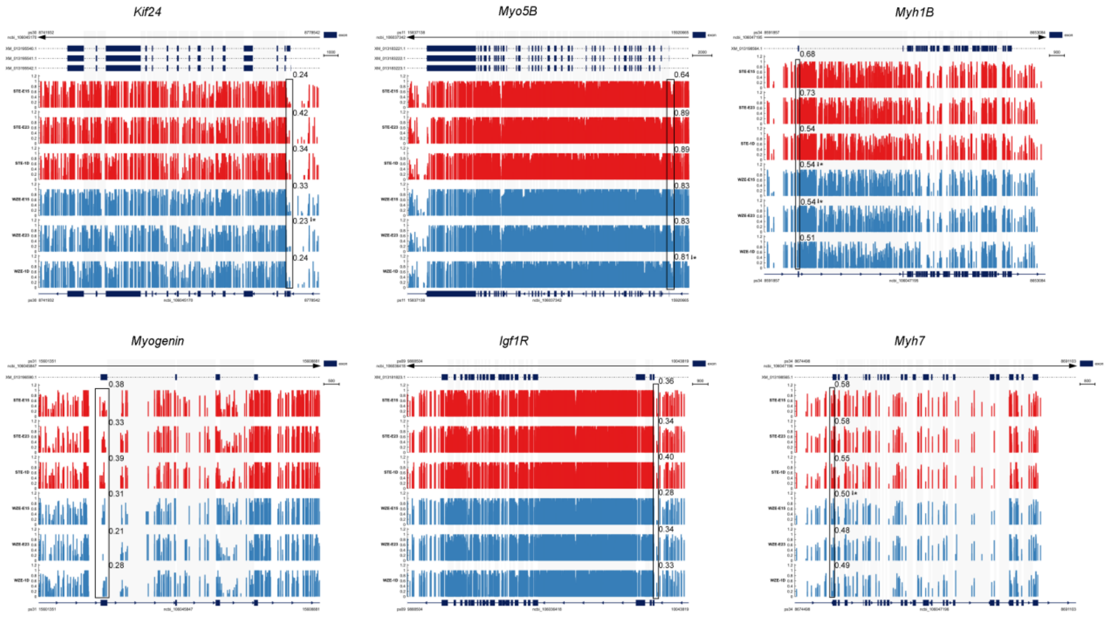

Figure S3. Methylation level of myogenic related DEGs related to DNA methylation or vital myogenic genes in WGBS data illustrated by IGV software. Regions around TSS are marked in black boxes with average DNA methylation level beside the boxes (n=3). Downward arrows indicate a lower DNA methylation level in comparison with STE.*$p < 0.05$.

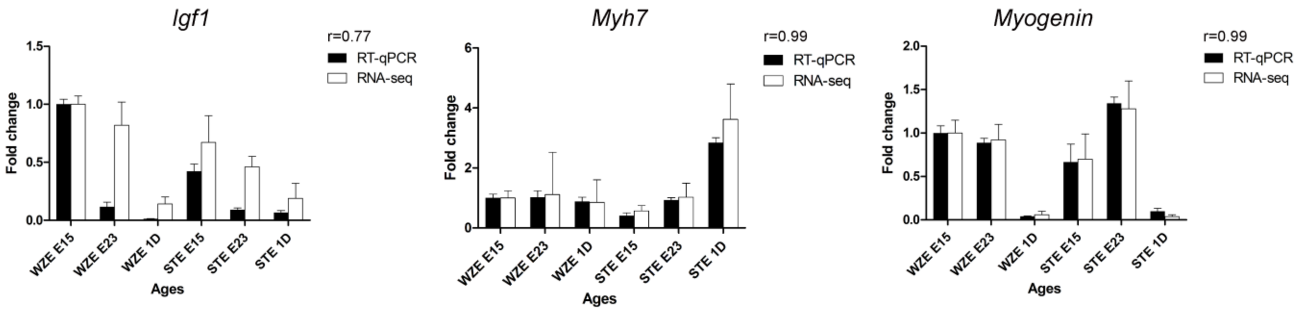

Figure S4. Validation of RNA-seq data by RT-qPCR.

RT-qPCR and RNA-seq validation of *Igf1*, *Myf7* and *Myogenin* expression (n=3). Pearson Correlation Coefficient (R) between RT-qPCR and RNA-seq was calculated.

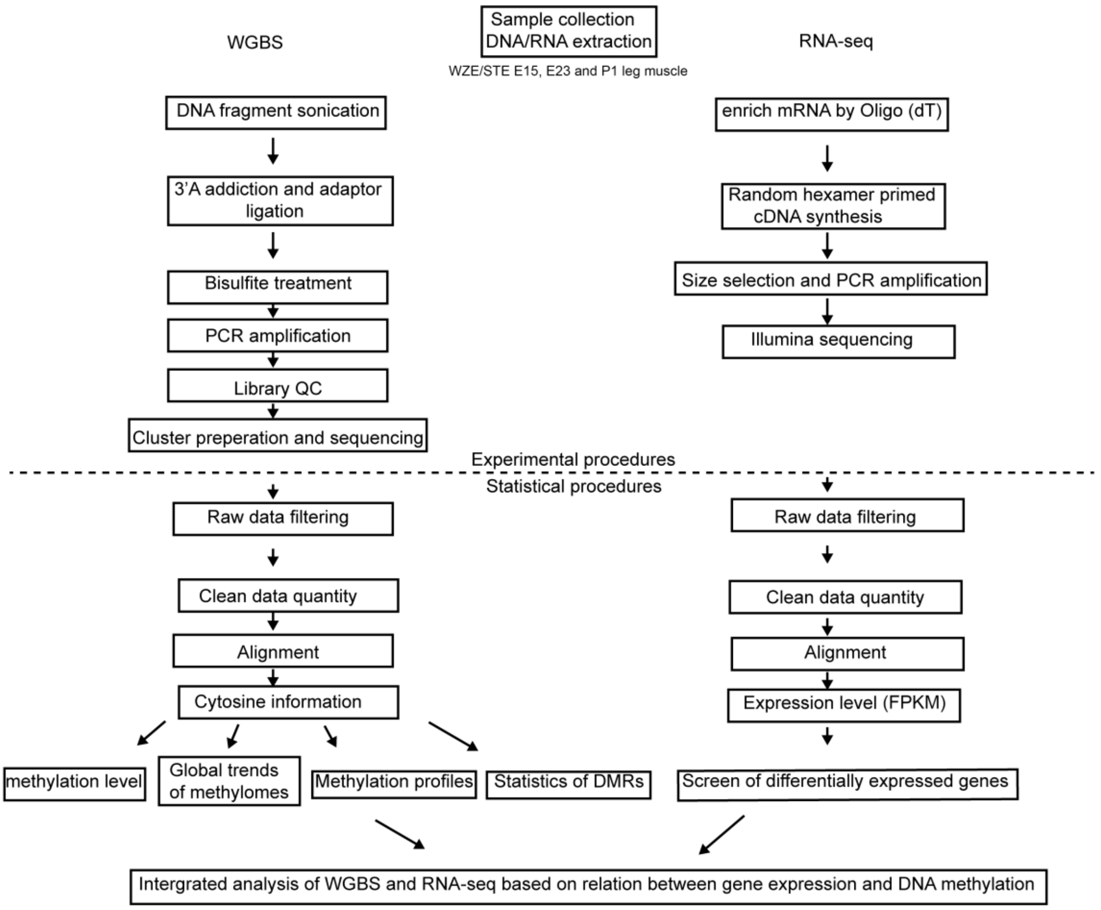

Figure S5. An overview of this study's design and analysis. This figure summarized sample collection, sample handling, experimental procedures, and data processing procedures.

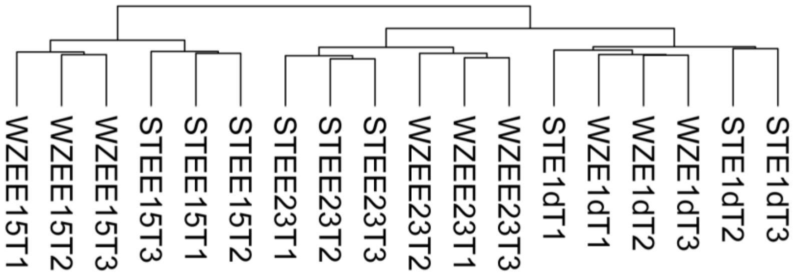

Figure S6. Cluster analysis of RNA-seq data. RNA-seq data of STE and WZE during E15 – P1 was used (n=3).
