# Supplementary material for: DNA Demethylation of Myogenic Genes May Contribute to Embryonic Leg Muscle Development Differences between Wuzong and Shitou Geese
Source: Int J Mol Sci. 2023 Apr 13;24(8):7188. doi: 10.3390/ijms24087188 (PMC10138404; doi:10.3390/ijms24087188)
Supplement: Supplementary file 1 [file ijms-24-07188-s001.zip › Table S6.pdf]

Table S6. The primers for RT-qPCR

Gene	Sequences
Igf1-F	AGGTCGTCCATCGTAGTCCTTGCA CTTTTAAGAAGCAATGGA
Igf1-R	ACAGCGTCGTTATCGTTCCTGC AAACACAGGCCAAGGTAG
Myog-F	CCCGAGCACTGCCCCGGGCAAT
Myog-R	CGCTCCTGCTGGTTGAGGCTGCTG
Myh7-F	GATGCGGAGATGGCCGCATT
Myh7-R	GACTTTGCCACCCTCTCGAGACA
Myod1-F	AAGGCGTGCAAGAGGAAGAC
Myod1-R	TGGTTGGGGTTGGTGGA
Tet1-F	GGAATGGAAGCCAAGATCAA
Tet1-R	ACTCCCTAAGGTTGGCAGTG
Tet2-F	CTGCTGTTTGGGTCTGAAGGAAGG
Tet2-R	GTTCTGCTGGTCTCTGTGGGAATG
Tet3-F	GCCGATGCAGTAGTGGAGG
Tet3-R	CTGCCTTGAATCTCCATGGTAC
Gapdh-F	TCTGTCGTGGACCTGACCTGC
Gapdh-R	GCCAGCACCCGCATCAAA
