# Supplementary material for: DNA Demethylation of Myogenic Genes May Contribute to Embryonic Leg Muscle Development Differences between Wuzong and Shitou Geese
Source: Int J Mol Sci. 2023 Apr 13;24(8):7188. doi: 10.3390/ijms24087188 (PMC10138404; doi:10.3390/ijms24087188)
Supplement: Supplementary file 1 [file ijms-24-07188-s001.zip › Table S7.pdf]

>ncbi_106033926

<--up2k--AACAACATTGTTACTTAAAAAAAAAAGGCAATTTATTGCACTTTT
TTGTTATTTAAAATTCTAAAAATAGAAAAAAGATCCCTTTATTTTTGTATATT
TGTAAATGTCCTGATGAAATTTCAAAACCAAAGCACTTAGCATACATAATTA
ACATACACATTTATGCCCTTTCTCTTTATCCATTGCAACAGTTTGAATAAGTT
TGAGTAAGATAAACCAATAGTGCTGGTTTCATCATGCAGCTTCAGCAGAGC
TTTCTGAAGCTACCAAATCAAACCAAAcgTTGCTAAGATACACACTTATCTT
CATGTTCAAGAGCAGCTAGTTTTCTTTAATTAGTAGTAGTTATCTGACTTTCT
GGAGGTAGCCTTCTGTAGGAAGCATCAGTTCTAAAACCTTCAGTGAAAAGT
TTCACACCTACATTGGGTCTGGCTCTGACTTGCcgTGTCAAGGCAGCTTGAT
AAGAGGAAGGGTCTGGTTTGCAGTGCTCCTCAGTTTTCCCTAAGCTCTGCA
TGTCAGTGCCATTGGCCACATTCAGATTTTTTCAATCTTcgTCCTGCAGAATA
TcgACTcgGAACCCATAGGCTATAAATcgATGGTTTCAGACTGTTGGTGTGTGT
CTGTAGACCCAGCTAGACTGCAGTTTGCCTTGCAGGGTGATCCCTAAATGG
GTTTTCAGGAGCCCAATTTAAGAACCAAAGTTTGCAAAcgCTGGGTCAGTC
TTATTTGTTCCTCAGCTCTGCTTATGTTCCTTGTCACAGTTGGAACTGAAAC
AAAGCTCAGAATCCAGATTTTTTCCAACTTTGAAATGTTTGCACATGGAcgG
GGTTGTACTTCcgTCAGCAACATGCCATCCTCTTGATGCACAAATGAGGAGA
GTAATTTCAGGCATTTATTTTAAAAATACTGCCTTAGAAATAGAcgGGCAAA
AATTGCACAGGGTATATATTTTTCTTATTTAAGTTGAAAAATAAATATAGCTG
TAATCTAAGTGTGTATTTATCAGCATTGATGCCCTTTGGTCTTGCTCCTGTGG
ACTTCTCTCACTGACATCAGTGGGAAAAAGATGGGTTCTGCACAGTAAATC
TGAATATTTTTTTCATCTTTATCCATCCATCTATTTTGGGATGTGCTAAATGCT
TGCTCTCTGTTGTAACATTAACATCAGTATGTGGAAACTTGGGTTGCTAAAA
TAAATCACTGAAATGCAATATACATGACTTCAcgTGGAACTGCATGTACATTG
TTGGGCACAAGCACCCTGcgGCcgTcgTGCcgTGCCAGCTGTAcgGcgGGGTGC
TTCCTATTGTGTCCTATAGAAGcgGGGTCTCAAAGTGAGACAGcgGCAGCTT
TGTGCTGCCAGTTTATATAAATAGTCTTTTTAGTCCCTGAGCTGGCAAAAGT
ATTTCTTGGTTGCCATTATcgTGATTGTGAATTTGAATCCATGATGCTTTTACC
AGGCTcgGAGTACAAAGAcgACAAGGTcgTGGTATTTCTCACAAGCATTTTTT

TCATTGTGcgCCTGTTTGTTTGCTGGAGGCAATGAAATAGTTATAATTTATAT

GTGAAGTACTGATTTGTGGGGCTCTAGCAGTGTTTCTTTAGTTTTTGAGTT

AAAGGTAGTGGCTTTGATCAAAATAGCATGGGGCATTTTTCTCAcgTTAGCA

GAGGCACTCCTGATTCATGACAGTATGAATGAGCACACCcgCAAGTTCATTT

CCTTTTTATATTTAAGGGAAAAAATGAAGGTAAATAAGAGGGGGAAAAGGG

GTAAGCTCAGCACTTACAACCTGGACTGTTTTCTGCCCACAGCAAAGTCAA

TGTGACACCTTTACAAAGCTGGAcgTTGATGAAGGTGTAGAGGGGACAGCC

CTGCCTCAGAGTCCCCTCcgATCTGcgCAGTGGTCCcgTGCCTCAGAGCACCA

GTTTGTCATCCACCATGGTCACCACACACAATGAGGGAGCTGATGAGCTGT

TCCCTTCTTACTTTGTCCTGCT--up2k--><--

1S

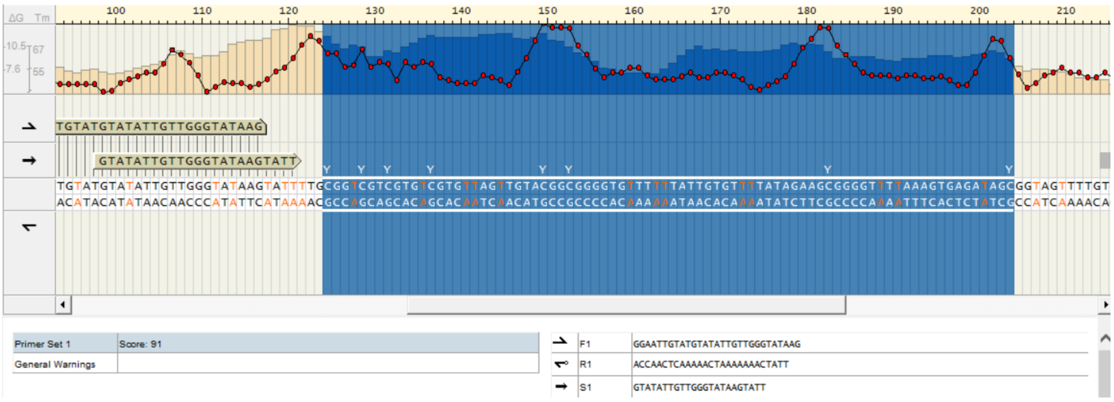

Primer Set 1	Score: 91
General Warnings	

F1	GGAATTGTATGTATATTGTTGGGTATAAG
R1	ACCAACTCAAAAACTAAAAAAACTATT
S1	GTATATTGTTGGGTATAAGTATT

	PCR Product	Forward PCR Primer, F1	Reverse PCR Primer, R1	Sequencing Primer, S1
Length, nt	170	29	27	23
Position, 5'- 3'		88 - 116	257 - 231	98 - 120
Warnings				
Tm, ºC		59.2	57.9	44.9
%GC	30.0	31.0	22.2	26.1
Sequence to Analyze	TTGYGGTYGT YGTGTYGTGT TAGTTGTAYG GYGGGGTGTT TTTTATTGTG TTTTATAGAA GYGGGGTTTT AAAGTGAGAT AGYGGTAGTT TTGTGTTGTT AGTTTATA			

Name: **Mispriming Analysis**

Description: Identifies alternate annealing sites for the sequencing primer on the amplicon.

Mispriming 1

```
5'- GTATATTGTTGGGTATAAGTATT -3'
         |    ||||   ||||
    3'- CCTTAACATACATATAACAACCCATATTCATAAAACGCCAGCAGCACAGCACAATCAACA... -5'

  Mispriming 3' Position: 104
  Penalty:                20
```

Mispriming 2

```
5'- GTATATTGTTGGGTATAAGTATT -3'
         |    ||||   ||||
    3'- CCTTAACATACATATAACAACCCATATTCATAAAACGCCAGCAGCACAGCACAATCAACA... -5'

  Mispriming 3' Position: 104
  Penalty:                20
```

Mispriming 3

```
                    5'- GTATATTGTTGGGTATAAGTATT -3'
                               | ||  |   ||||| || |
3'- ...CACTCTATCGCCATCAAAACACAACAATCAAATATATTTATCAAAAAAATCAAAAAC... -5'

  Mispriming 3' Position: 236
  Penalty:                15
```

Penalty: 20

2S

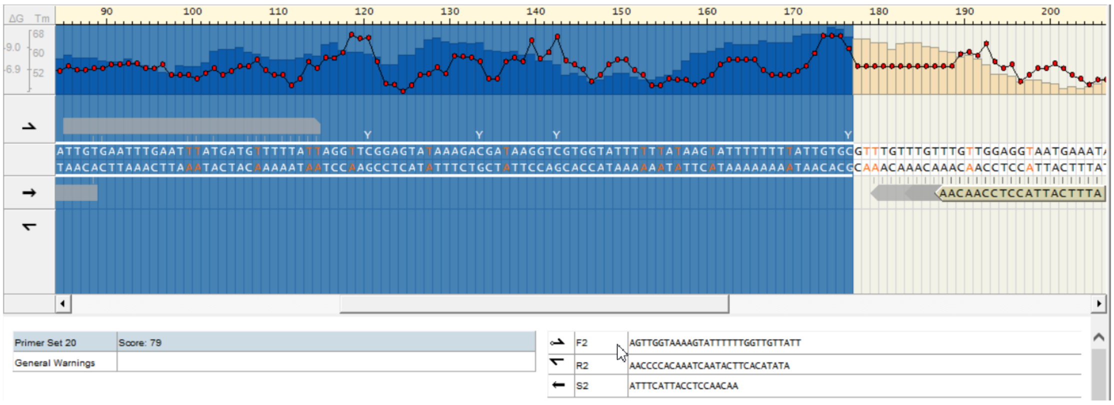

Primer Set 20	Score: 79
General Warnings	

→	F2	AGTTGGTAAAAGTATTTTTTGGTTGTTATT
←	R2	AACCCCACAAATCAATACTTCACATATA
←	S2	ATTTCATTACCTCCAACAA

	PCR Product	Forward PCR Primer, F2	Reverse PCR Primer, R2	Sequencing Primer, S2
Length, nt	196	30	28	19
Position, 5'- 3'		48 - 77	243 - 216	205 - 187
Warnings				
Tm, ºC		59.9	61.7	45.2
%GC	24.5	23.3	32.1	31.6
Sequence to Analyze	ACAAACAAAC RCACAATAAA AAAAATACTT ATAAAAAATA CCACRACCTT ATCRTCTTTA TACTCCRAAC CTAATAAAAA CATCATAAAT TCAAATTCAC AATCACRATA ATAACAACCA AAAAATACTT TT			
